# Predictive algorithms for determination of reflectance data from quantity of pigments within experimental dental resin composites

**DOI:** 10.1186/1475-925X-14-S2-S4

**Published:** 2015-08-13

**Authors:** Razvan Ghinea, Oscar Pecho, Luis Javier Herrera, Ana Maria Ionescu, Juan de la Cruz Cardona, María Purificación Sanchez, Rade D Paravina, María del Mar Perez

**Affiliations:** 1 Department of Optics, University of Granada, Campus Fuentenueva s/n, 18071, Granada, Spain; 2 Department of Computer Architecture and Computer Technology, University of Granada, C/ Periodista Daniel Saucedo Aranda s/n, 18071, Granada, Spain; 3 Department of Inorganic Chemistry, University of Granada, Campus Fuentenueva s/n, 18071, Granada, Spain; 4 Houston Center for Biomaterials and Biomimetics, Department of Restorative Dentistry and Prosthodontics, University of Texas, School of Dentistry, 7500 Cambridge St., Houston, TX 77054, USA

**Keywords:** Experimental dental composites, predictive algorithms, reflectance, color coordinates

## Abstract

**Background:**

Being able to estimate (predict) the final spectrum of reflectance of a biomaterial, especially when the final color and appearance are fundamental for their clinical success (as is the case of dental resin composites), could be a very useful tool for the industrial development of these type of materials. The main objective of this study was the development of predictive models which enable the determination of the reflectance spectrum of experimental dental resin composites based on type and quantity of pigments used in their chemical formulation.

**Methods:**

49 types of experimental dental resin composites were formulated as a mixture of organic matrix, inorganic filler, photo activator and other components in minor quantities (accelerator, inhibitor, fluorescent agent and 4 types of pigments). Spectral reflectance of all samples were measured, before and after artificial chromatic aging, using a spectroradiometer. A Multiple Nonlinear Regression Model (MNLR) was used to predict the values of the Reflectance Factors values in the visible range (380 nm-780 nm), before and after aging, from % Pigment (%P1, %P2, %P3 and %P4) within the formulation.

**Results:**

The average value of the prediction error of the model was 3.46% (SD: 1.82) across all wavelengths for samples before aging and 3.54% (SD: 1.17) for samples after aging. The differences found between the predicted and measured values of the chromatic coordinates are smaller than the acceptability threshold and, in some cases, are even below the perceptibility threshold.

**Conclusions:**

Within the framework of this pilot study, the nonlinear predictive models developed allow the prediction, with a high degree of accuracy, of the reflectance spectrum of the experimental dental resin composites.

## Background

It has been reported that the composite restorative materials are one of the many successes of modern biomaterials research due to their capability to replace biological tissue in both appearance and function[1]. In a recent review of treatment considerations for esthetic restorations it has been pointed out that at least half of the posterior direct restoration placement now rely on composite materials[2]. Currently, methacrylate resin formulations dominate both the commercial market and research studies. The resin phase is composed primarily of dimethacrylate monomers (typically selected from Bis-GMA, BisEMA, and/or UDMA or a mixture of them) usually mixed with a low-viscosity reactive diluents (most commonly TEGDMA). These base monomers result in restorative materials with excellent mechanical properties, rapid polymerization, and low shrinkage.

Several authors implemented the use of experimental dental composites, as a continuous effort to understand the interrelationships among composition, resin viscosity, degree of conversion, shrinkage, flexural strength, fracture toughness, water sorption and solubility, etc. This type of materials were used to study the physical and mechanical properties of a new methacrylate monomer through comparisons with a commonly used Bis-GMA monomer[3], the effects of ceramic and porous fillers on the mechanical properties[4], the influence of irradiant energy on the degree of conversion, polymerization rate and shrinkage stress[5], the effect of co-initiator ratio on the polymer properties[6] or the curing efficiency of dental resin composites[7]. Furthermore, the use of a fluorescent agent was incorporated in the chemical formulation to study the influence of the fluorescent whitening agent on the fluorescent emission of resin composites[8].

Color prediction in dentistry is a research area that has barely been explored. So far it has been proven that the polymerization dependent color changes in resin composites can be successfully predicted using multivariable linear models of statistical inference[9]. Also, in a recent study, the color change after tooth bleaching was predicted using a novel fuzzy logic model[10] while other authors were able to predict the final color of 25 opaque feldspathic dental ceramic specimens fabricated by mixing six different pure shades in different concentrations[11].

The reflectance spectra is a physical characteristic of a sample, which provides valuable information with respect to the interaction of light (incident radiation) with the sample. Furthermore, based on the values of the reflectance factors for each wavelength of the visible spectrum, the final color of the sample under any available illuminant can be calculated. Therefore, being able to estimate (predict) the spectrum of reflectance of a biomaterial, especially in the case of materials whose color and appearance are fundamental for its clinical success (as it is the case of dental resin composite), could be a very useful tool for the industrial development of these type of materials. However, to the best of our knowledge, there is no available study on reflectance predictions for experimental dental resin composites.

Therefore, the main objective of this study is the development of predictive models which enable the determination of the reflectance spectrum in the visible range of experimental dental resin composites using as input data the type and quantity of pigments used in their chemical formulation.

## Methods

### Experimental dental resin formulation

For the development of this study, 49 different types of experimental dental resin composites (n = 3) were formulated as a mixture of organic matrix, inorganic filler, photo activator and other components in minor quantities: accelerator, inhibitor, fluorescent agent and four types of pigments (in various mixtures) according to available standards and literature[8], and following standard manufacturing procedures. The relative quantities of each chemical component within the experimental resin composites are listed in Table 1.

**Table 1:** Chemical components and their relative percentages by weight (% w/t) within the total mixture used to formulate the resin composites from this study.

Component		Chemical Name		% w/t
Organic Matrix	45% BisGMA	Bisphenol A glycerolatedimethacrylate		68.8% (Average)
		
	45% TEGDMA	Triethylene glycol dimethacrylate		
		
	10% UDMA	DiurethaneDimethacrylate		

Inorganic Filler		SiO_2 _Glass Spheres [Φ: 9-13µm]		30%

Photo Activator		Camphorquinone		0.7%

Accelerator		2-(Dimethylamino)ethyl methacrylate		0.35%

Inhibitor		Butylatedhydroxytoluene		0.05%

Fluorescent Agent		1.4-Bis(2-benzoxazolyl)naphthalene		0.04%

Pigment		Pigment 1 (P1)	FeO·OH	0.06% (Average)
		
		Pigment 2 (P2)	FeO	
		
		Pigment 3 (P3)	TiO_2_	
		
		Pigment 4 (P4)	Fe_2_O_3_	

A total of 49 different mixtures of pigments were formulated by varying the relative amount of each of the four pigments. All chemical components were weighted using a high precision digital scale (BL60S, Sartorius AG, Goettingen, Germany) and carefully hand-mixed until a homogeneous mixture was obtained. All specimens were cylinder shaped with a diameter of 20 mm and 1.5 mm thickness. Each of the 147 specimens was packed in a custom built silicon mold in a glass plate sandwich with a mylar strip on both sides. The specimens were light cured at 1100 mW/cm^2 ^for 60 seconds on each side using a LED light-curing unit (BluePhase, Ivoclar Vivadent AG, Schaan, Liechtenstein). In order to mimic a clinical situation and before storage, all samples were polished using a one-step diamond micro-polishing system (PoGo, Dentsply, USA), by applying light intermittent pressure at moderate speed during 40s.

All samples underwent an artificial chromatic aging. Specimens were placed inside an artificial aging chamber (Suntest XXL, ATLAS, USA) and subjected to artificial chromatic aging. The artificial aging cycle was defined as a 102 minutes dry and 18 minutes of water spray both under an artificial daylight simulator equivalent to CIE D65 Illuminant at 38 ± 3°C constant temperature and 50 ± 10% relative humidity, as described by ISO 4892-2 A1 and ISO 7491 Standards[12,13].

### Reflectance measurements

The reflectance spectrum in the 380 nm-780 nm range of all specimens was measured inside a completely dark room using a spectroradiometer (PR 670, PhotoResearch, USA) and a spectrally calibrated reflectance standard (SRS-3, PhotoResearch, USA). Specimens were placed on a custom built sample holder, 40 cm away from the spectroradiometer and illuminated using a Xe-Arc Light Source (Oriel Research, Newport Corporation, USA). The illuminating/measuring geometry corresponded to CIE 45°/0°. The aperture of the spectroradiometer was set to 1°, which allowed the measurement of a central spot (measuring field) of the specimen of approximately 0.7 cm.

Short-term repeated reflectance measurements without replacement were performed and each sample was measured three times. As three samples of each group were produced, this gave a total of 9 recording for each type and the results were averaged. This procedure was performed before and after the chromatic artificial aging procedure.

### Color and color difference calculations

Color calculations (chromatic coordinates CIE L^*^, a^* ^and b^*^) for both real and predicted reflectance spectra were performed in base of the CIE D65 Standard Illuminant and the CIE Colorimetric 2° Standard Observer assumptions[14].

The total color differences between the predicted and the measured (real) values of the CIE L^*^a^*^b^* ^chromatic coordinates were calculated according to the CIELAB color difference formula ($\text{Δ}E_{ab}^{*}$):

$$\text{Δ}E_{ab}^{*}=\sqrt{\text{Δ}{L^{*}}^{2}+\text{Δ}{a^{*}}^{2}+\text{Δ}{b^{*}}^{2}}$$

and the CIEDE2000 total color difference formula (Δ*E*_00_), which corrects for the non-uniformity of the CIELAB color space for small color differences under reference conditions:

$$\text{Δ}E_{00}\sqrt{{\left(\frac{\text{Δ}L^{\prime }}{K_{L}S_{L}}\right)}^{2}+{\left(\frac{\text{Δ}C^{\prime }}{K_{C}S_{C}}\right)}^{2}+{\left(\frac{\text{Δ}H^{\prime }}{K_{H}S_{H}}\right)}^{2}+R_{T}\left(\frac{\text{Δ}C^{\prime }}{K_{C}S_{C}}\right)\left(\frac{\text{Δ}H^{\prime }}{K_{H}S_{H}}\right)}$$

where Δ*L'*, Δ*C' *and Δ*H' *are the differences between the two samples forming the pair in lightness, chroma and hue, and *R_T _*is the rotation function which accounts for the interaction between chroma and hue differences in the blue region. The weighting functions *S_L_, S_C _*and *S_H _*adjust the total color difference for variation in the location of the color difference pair in *L', a'*and *b' *coordinates and the parametric factors *K_L_, K_C _*and *K_H _*are correction terms for experimental conditions. For calculations made in this study, all the parametric factors were set to 1. All the discontinuities due to mean hue computation and hue-difference computation pointed out and characterized by Sharma and collaborators[15] were taken into account when the calculations with the CIEDE2000 color difference formula were performed.

### Design of the predictive models

Samples were divided into a Training (Active) Group, which contained 44 samples (sample 1 to 44) from the total of 49 samples available, and a Testing (Validation) Group, which contained 5 samples from the total of 49 samples available (Sample 45 to 49). Samples in the Testing Group present a combination of the 4 pigments. In all cases, the Active (Training) Group was used to build the predictive model while the Validation Group was used exclusively for testing the appropriate functioning of the model as well as its accuracy.

A Multiple Nonlinear Regression Model (MNLR) was used to predict the values of the Reflectance Factors values in the visible range (380 nm-780 nm) before and after aging from % Pigment (%P1, %P2, %P3 and %P4) within the formulation.

The equation describing the model is a 4th Order Polynomial, as described by:

$$\begin{array}{c} Y=pr1+pr2\cdot X_{1}+pr3\cdot X_{2}+pr4\cdot X_{3}+pr5\cdot X_{4}+pr6\cdot X_{1}^{2}+pr7\cdot X_{2}^{2}+pr8\cdot X_{3}^{2}+pr9\cdot X_{4}^{2}+\\ +pr10\cdot X_{1}^{3}+pr11\cdot X_{2}^{3}+pr12\cdot X_{3}^{3}+pr13\cdot X_{4}^{3}+pr14\cdot X_{1}^{4}+pr15\cdot X_{2}^{4}+pr16\cdot X_{3}^{4}+pr17\cdot X_{4}^{4} \end{array}$$

where:

- Y is the predicted variable: Reflectance Factors values in the visible range (380 nm-780 nm) at 2 nm step the before and after aging;

- X_i _are the input variables: %P1, %P2, %P3 and %P4;

- pr1 ... pr17 are the parameters of the model.

The models were built using the Training Group and tested using the Validation Group. The model was considered to be accurate after 200 iterations were performed and/or a convergence of 0.00001 was achieved.

A total of 402 models (one for each Reflectance Factor before and after aging) were designed. All the Multiple Nonlinear Regression predictive models were designed using a commercial software (XLSTAT, Addinsoft, USA).

## Results and discussion

In dentistry, it would be ideal to achieve a restoration that has identical colors as the tooth structure under various illumination conditions, within at least acceptable limits, but more preferably within limits of perceived color difference[16]. Although the importance of the pigments on the final color and appearance of the dental resin composite is well known, there is no research study available which made use of coloring pigments when formulating the experimental dental resin composites. Therefore, in this study, the use of pigments was included in the chemical formulation of the resin composites.

The research in science and biomedical applications often involves using controllable and/or easy to measure variables (input factors) to explain, regulate or predict the behavior of other variables (output factors or dependent variables). When dealing with a reduced number of input factors which are not significantly redundant and have a strong relationship with the output variables, the MNLR is one of the best options to take into account for modeling the data.

**Figure 1:**
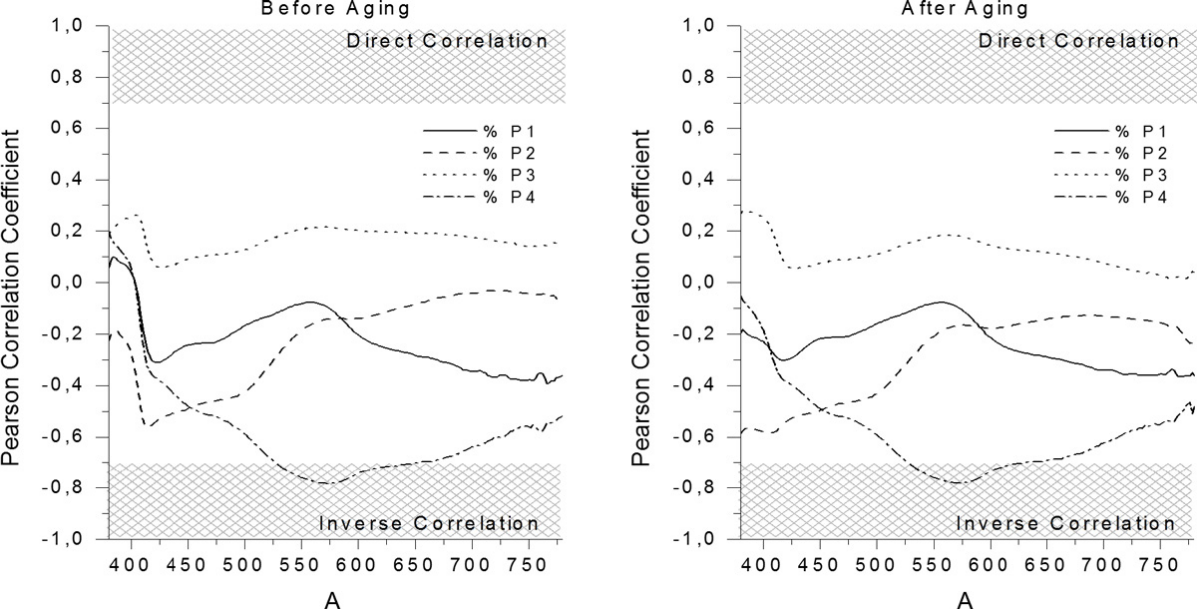
**Pearson Correlation Coefficients between 380 nm-780 nm Reflectance Factors before and after aging and % Pigment**.

The correlation between the input variables of the model (in this case, the Reflectance Factors at 2 nm steps in the 380 nm-780 nm interval before and after the artificial aging) and the output variables of the model (in this case, the percentage of each type of Pigment used - %P1, %P2, %P3 and %P4), was carried out as an initial step. The results obtained for the Pearson Correlation coefficient are graphically presented in Figure 1.

The correlation between variables, when biomaterials are studied, is considered to be strongly direct if the value of the Pearson Coefficient exceeds 0.7 and is considered to be strongly inverse if the value of the Pearson Coefficient is lower than -0.7. As it can be observed in Figure 1, for the 5 samples included in the testing group (Validation Group) before aging, there is a strong inverse correlation between the quantity of the fourth Pigment (%P4) and reflectance factors of wavelengths between 525 nm-650 nm. This implies that higher quantities of Pigment 4 within the mixture of the experimental resin composite will decrease the values of the reflectance factors between the specified interval, affecting the lightness value of the sample and generating an orange-reddish color shift. Also, a strong correlation usually is associated with increased performance of the predictive model, but no conclusion should be drawn before analyzing the other parameters of the quality of fit, such as goodness of fit or relative residuals.

**Figure 2:**
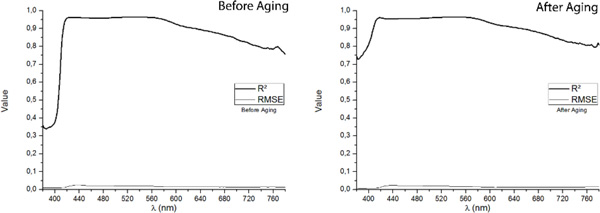
**Goodness of fit (in terms of R^2 ^and RMSE) for the Reflectance Factors (380 nm-780 nm) for samples before aging (left) and after aging (right)**.

The goodness of fit, in terms of R^2 ^and the Root Mean Square Error (RMSE) for the predictive models of the Reflectance Factors for wavelengths between 380 nm-780 nm, both before and after the artificial aging, is displayed on Figure 2. Numerical measures of goodness of fit are divided into two types: measures of deviation from the real (measured) values and measures of how well the trend relative magnitudes are predicted. If only one type of these measurements is used, only one of these two types of information is being captured, and that it is why several researchers recommend the use of a combination of R^2 ^for trend relative magnitude and RMSE for deviation from exact data location[17]. We found high values (>0.7) of the Coefficient of Determination for the predictive models of the Reflectance Factors for wavelengths higher than 425 nm, both before and after aging. However, it seems that the predictive model works best for wavelengths between 425 nm and 600 nm, since in this interval the R^2 ^values are higher than 0.9. This mean that future works should be focused on improving the MNLR models in order to obtain better performance for larger wavelengths. It should be noted that, if we assess the quality of the predictive model on the exclusive basis of the value of R^2^, the model performs better for aged samples, since the values obtained for the Coefficient of Determination are slightly higher. Both before and after aging, the RMSE values are very low, in accordance to the interval of the studied variable. All these results support the quality of the Multiple Nonlinear Regression Predictive model designed and serve to ensure the proper development of the method.

**Figure 3:**
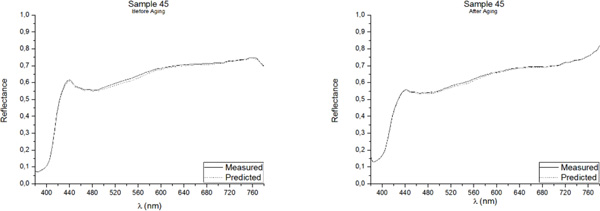
**Real (measured) and Predicted spectral reflectance of Sample 45 between 380 nm-780 nm before aging (left) and after aging (right)**.

**Figure 4:**
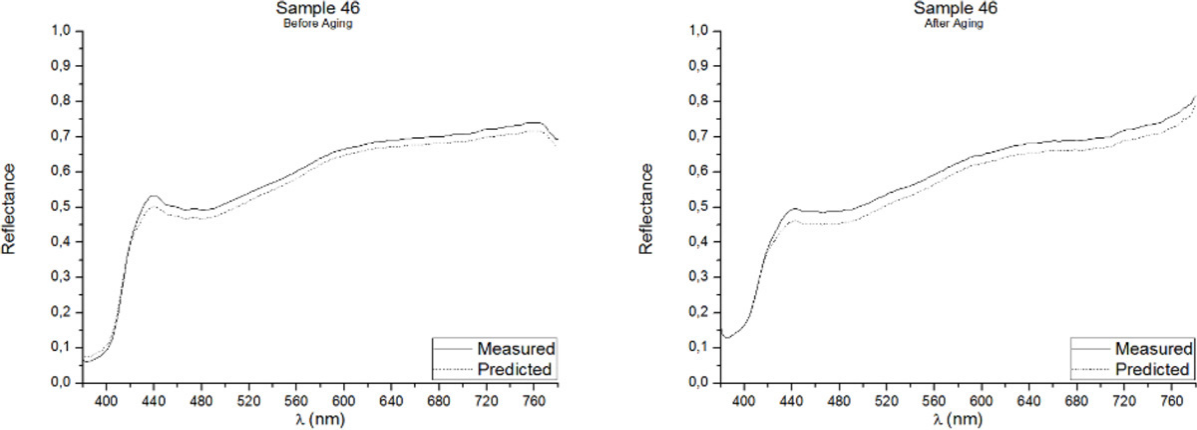
**Real (measured) and Predicted spectral reflectance of Sample 46 between 380 nm-780 nm before aging (left) and after aging (right)**.

**Figure 5:**
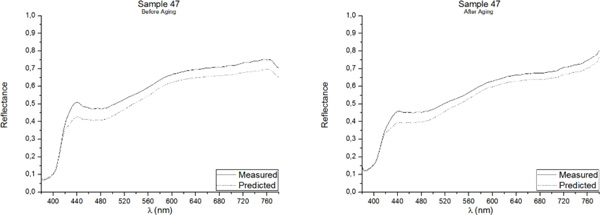
**Real (measured) and Predicted spectral reflectance of Sample 47 between 380 nm-780 nm before aging (left) and after aging (right)**.

**Figure 6:**
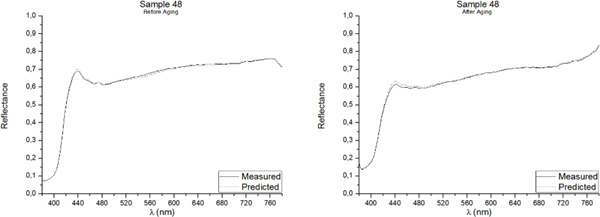
**Real (measured) and Predicted spectral reflectance of Sample 48 between 380 nm-780 nm before aging (left) and after aging (right)**.

**Figure 7:**
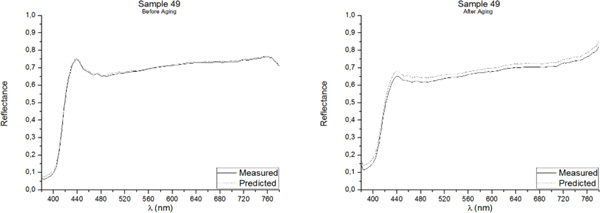
**Real (measured) and Predicted spectral reflectance of Sample 49 between 380 nm-780 nm before aging (left) and after aging (right)**.

One of the best methods for assessing the accuracy of point predictions is to use overlay scatter plots and overlay line graphs. In these graphical forms, the model and data are overlaid on the same graph, allowing a direct comparison of the real (measured) data and the predicted values. The reflectance spectrum of the five samples included in the Validation Group, as measured with the PR-670 Spectrorradiometer and as predicted with the Multiple Nonlinear Regression model, both before and after aging, are presented in Figures 3, 4, 5, 6, 7. Visual displays of goodness of fit are useful for a rough estimate of the degree of fit and for indicating where the fits are most problematic. Visual displays are also useful for diagnosing a variety of types of problems (e.g., systematic biases in model predictions). Noteworthy, the human visual system is not particularly accurate in assessing small to moderate differences in the fits of model to data.

Our visual system is also subject to many visual complications that can produce systematic distortions in the visual estimates of the quality of a fit[17]. However, as it can be observed in Figures 3, 4, 5, 6, 7, the quality of the fit is excellent for almost the entire spectrum, providing accurate estimates of the Reflectance Factors for all wavelengths.

**Figure 8:**
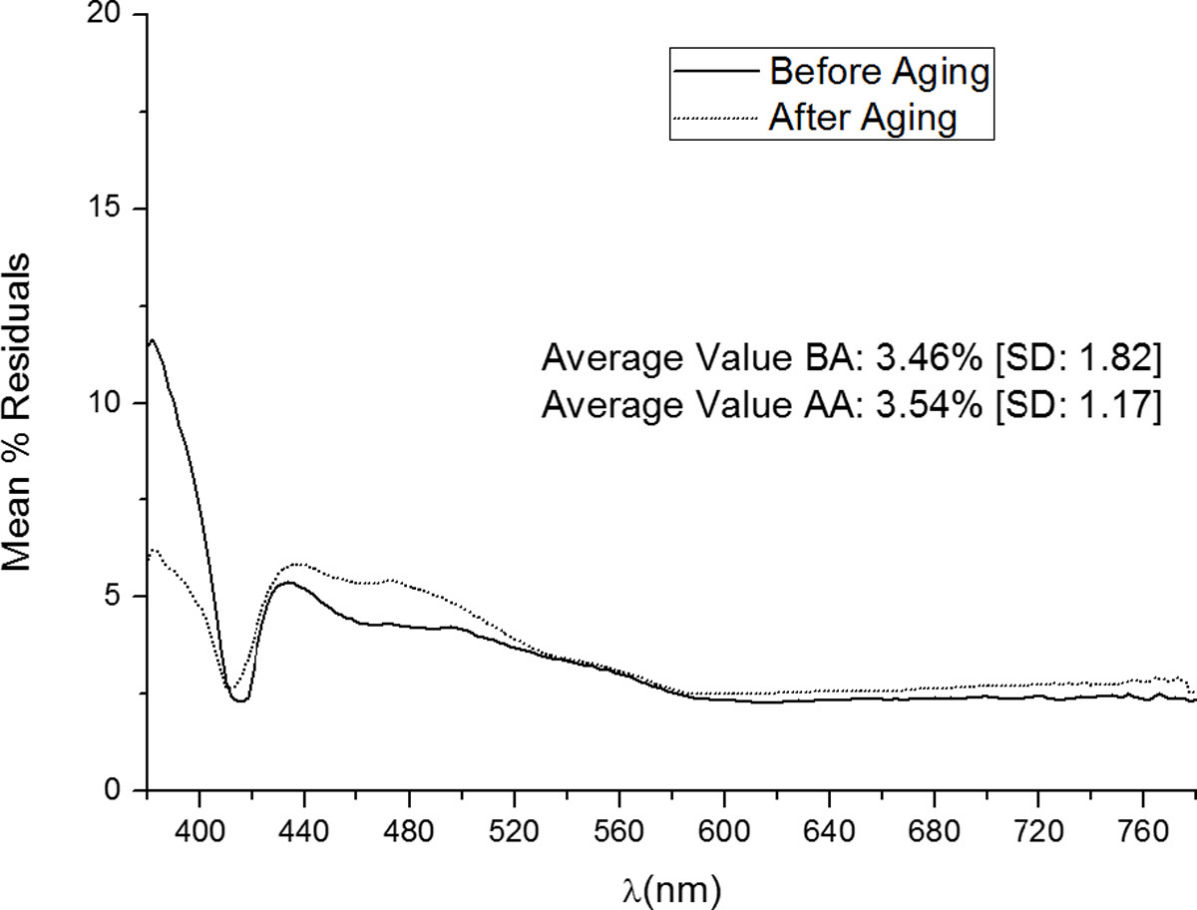
**Mean %Residuals of all samples of the Validation Group, before and after aging, in the 380 nm-780 nm interval**.

In order to assess the overall quality of the prediction capacity of the proposed models, the mean value over the 5 samples (relative to the value of the variable - Reflectance Factor) along the 380 nm-780 nm interval was calculated. The results for samples before and after the aging procedure was applied are schematically shown in Figure 8. For samples before aging, the average value of the prediction error of the model was 3.46 ± 1.82%, showing higher values for shorter wavelengths and considerably lower values for longer wavelengths In the case of the samples after aging, the average value of the prediction error was 3.54 ± 1.17%, exhibiting, as the case of samples before aging, lower error for longer wavelengths. The high errors obtained for short wavelengths are probably caused by the instability of the measuring system (the spectrorradiometer) which presents variability in the measured data for wavelengths lower than 400 nm. This variability is expected to affect the quality of the predictive model, since no clear pattern in the input data can be established, so the provided output variables are distant from the measured ones.

Several MNLR predictive models have been developed, which accurately predicted the reflectance spectrum of the manufactured experimental dental resin composites. These models are very helpful when, in a laboratory situation, the chromatic behavior of the samples needs to be controlled. In this study, we considered the pigments as the main responsible (not exclusive) of the final color of the composites, and therefore we centered the study on the influence of the four types of pigments on the final color of the experimental dental resin composites.

**Figure 9:**
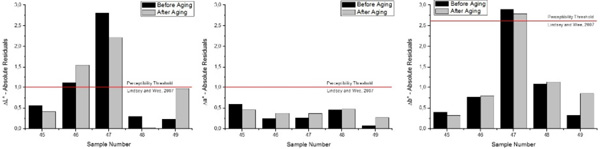
Absolute Residual values of CIELAB coordinates (L^*^, a^* ^and b^*^) before and after aging.

In Figure 9 are plotted the absolute values of the Residuals for CIE L^*^, a^* ^and b^* ^coordinates before and after aging. Among the three chromatic coordinates studied, for all samples included in the Validation Group, the CIE a^* ^coordinate showed the smallest residuals, with absolute residual values smaller than 0.6 in all cases. When analyzing absolute residuals, the magnitude of the studied variable it must be taken into account, since the a^* ^chromatic coordinate vary within a scale of 2-6 units, while the b^* ^coordinate can reach double superior values and the L^* ^coordinate values are within the 80-85 units range. Therefore, obtaining very low values of residuals for this particular variable it is not so surprising, and the differences between the predicted and the measured values must be evaluated from a more objective point of view, such as comparisons with the chromatic perceptibility thresholds in dentistry. In the case of the yellow-blue axis of the color space (b^*^), the predicted values matched very closely the measured ones. The smallest residual registered was 0.319, while the greatest one was 2.787. In the case of the L^* ^chromatic coordinate the absolute differences between the measured and the predicted values ranged between 0.232 and 2.811 units.

In a study on the perceptibility and acceptability of tooth color differences using computer-generated pairs of teeth with simulated gingival displayed on a calibrated monitor, Lindsey and Wee[18] established that ΔL* = 1.0, Δa* = 1.0 and Δb* = 2.6 units are considered as 50:50% perceptibility threshold for the human eye. In our study, the differences between the predicted and the measured values of the chromatic coordinates exceeded the lightness threshold for 40% of the studied samples (both before and after aging). For the b* chromatic coordinate, 80% of the samples presented differences which were unable to be perceived by a human observer with normal color vision, while for the red-green axis (a* coordinate) all the studied samples exhibited differences bellow the perceptibility threshold for this coordinate, independently if the sample were analyzed before or after the aging procedure was applied.

**Figure 10:**
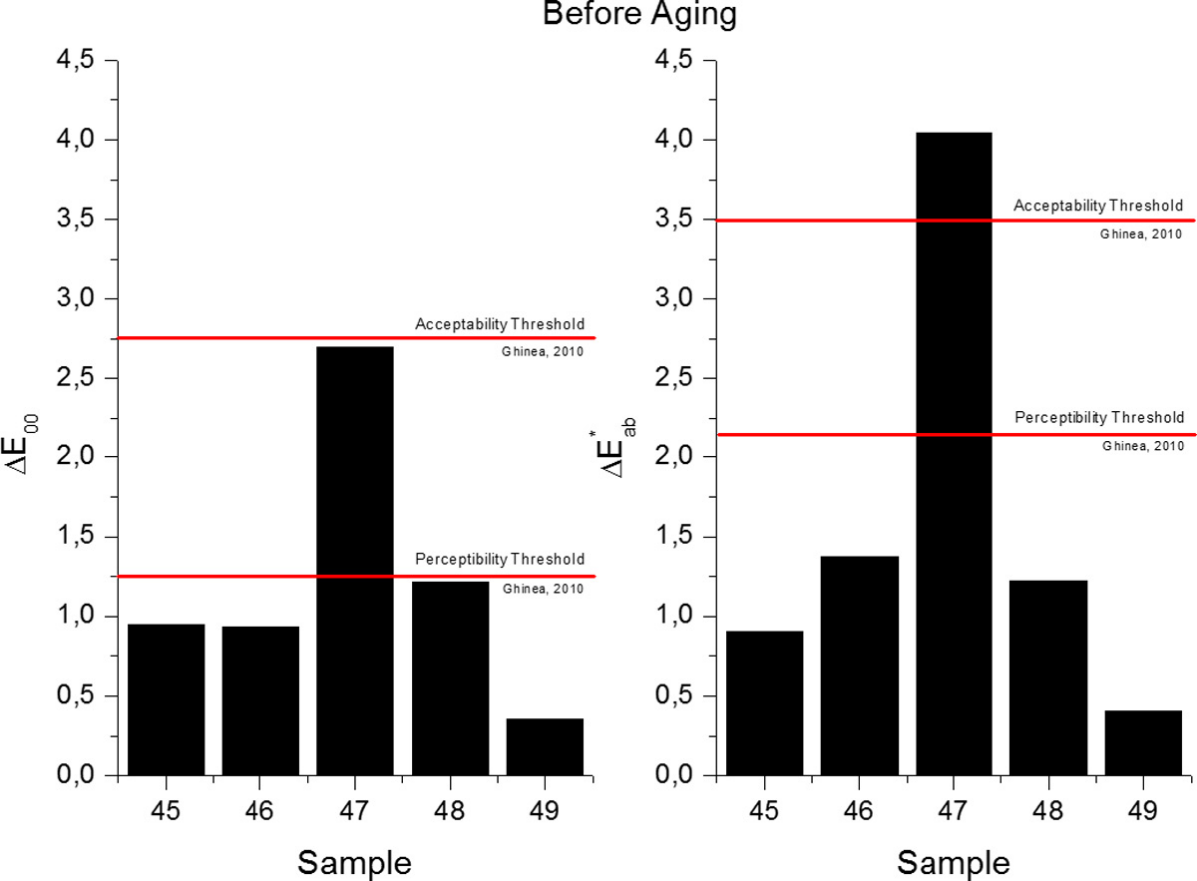
Total color differences in terms of Δ*E*_00 _(left) and $\text{Δ}E_{ab}^{*}$ (right) between the predicted and measured values for samples in the Validation Group before aging.

**Figure 11:**
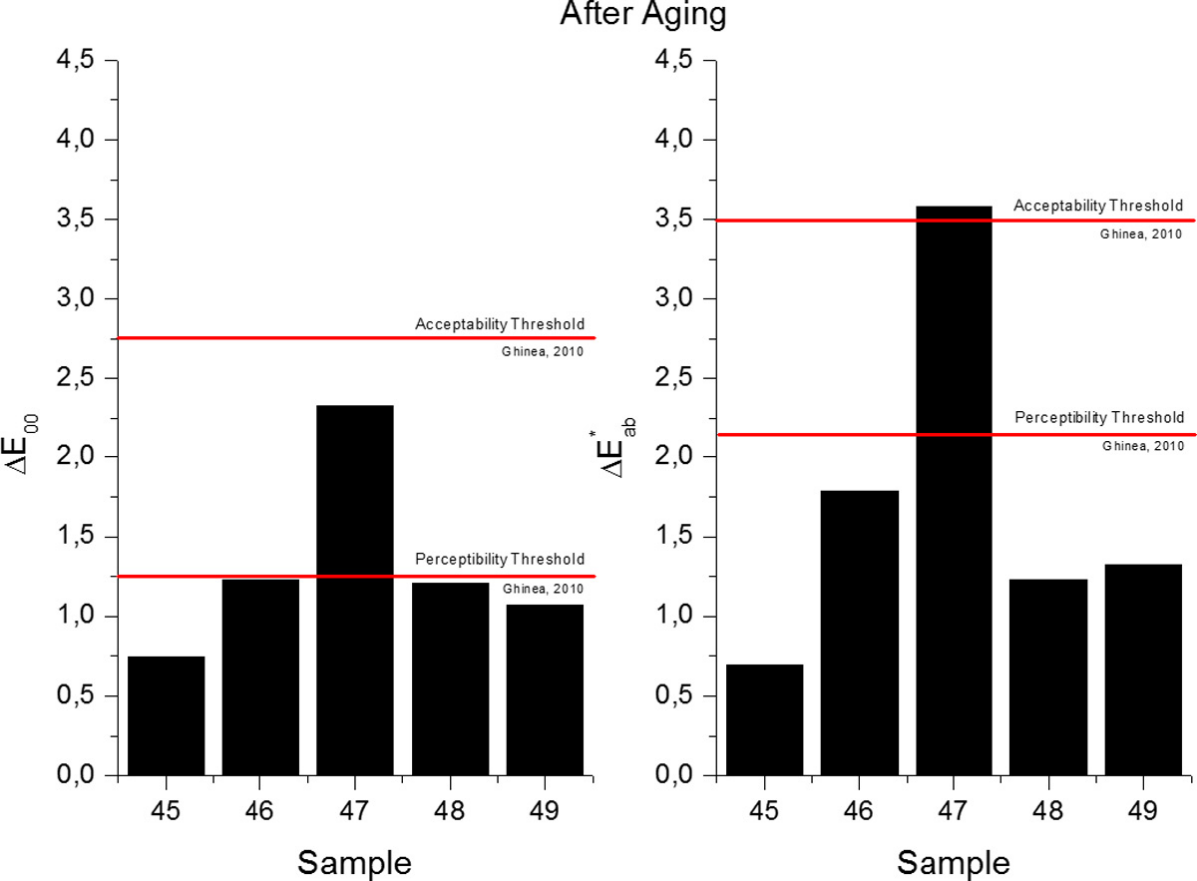
Total color differences in terms of Δ*E*_00 _(left) and $\text{Δ}E_{ab}^{*}$ (right) between the predicted and measured values for samples in the Validation Group after aging.

The study on the accuracy of the MNLR predictive models can be extended with the calculation of the total color differences (in terms of Δ*E*_00 _and $\text{Δ}E_{ab}^{*}$ total color differences), between the predicted and measured values of the chromatic coordinates, and consequently evaluate them through comparisons with the available perceptibility and acceptability thresholds for dentistry[19]. The total color differences in terms of Δ*E*_00 _and $\text{Δ}E_{ab}^{*}$ between the predicted and measured values for the samples included in the Validation Group before aging are presented in Figure 10 while the values obtained for samples after aging are presented in Figure 11. In the case of Sample 47, the differences found were higher than both thresholds, probably due to the high discrepancies between the predicted and measured value of lightness (L^*^). After the artificial aging procedure was applied, if the differences between the predicted and measured values are computed with the newest Δ*E*_00 _formula, all values fall within the acceptability threshold, and in 75% of the studied cases, the differences are even smaller than the perceptibility threshold. If, instead, the CIE1976 total color difference formula is used ($\text{Δ}E_{ab}^{*}$), for Sample 47, similar to what happens before aging, the value of the difference is higher than both thresholds, while for the other 4 samples included in the Validation Group, the calculated total color differences are smaller than both perceptibility and acceptability thresholds.

**Figure 12:**
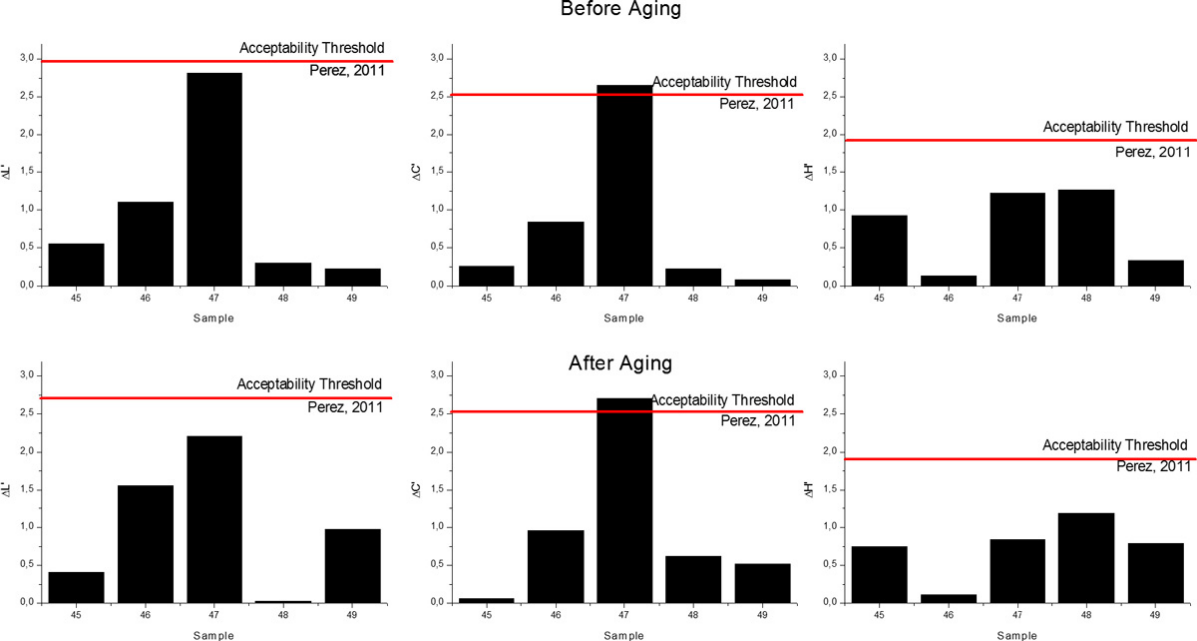
**Absolute values of the differences in lightness (Δ*L'*), chroma (Δ*C'*) and hue (Δ*H'*) between predicted and measured values for the Validation Group before aging (top) and after aging (bottom)**.

The absolute values of the differences in lightness (Δ*L'*), chroma (Δ*C'*) and hue (Δ*H'*) between the predicted and the measured values for the five samples included in the validation group (both before and after aging) are presented in Figure 12.

The visual 50:50% acceptability thresholds for lightness, chroma and hue in dentistry were established as Δ*L' *= 2.92, Δ*C' *= 2.52 and Δ*H' *= 1.90 units[20]. The differences found between the predicted and the measured values are smaller than the acceptability thresholds for lightness and hue, independently if the samples are analyzed before or after the aging procedure was applied. In the case of chroma differences, for one of the samples included in the Validation Group, the computed difference exceeded the acceptability threshold, while all the other four samples exhibited values considerably lower than the threshold.

It has to be mentioned that the range of application of the proposed predictive models is limited, since they are designed to work exclusively with the experimental dental resins developed in this study. It is necessary to expand the present work with further studies on multiple areas, such as varying the materials used for the formulation, varying amounts of both the organic matrix and the inorganic filler as well as the quantities of the other components used in the chemical formulation.

It would also be interesting to study more carefully the behavior of the different pigments, through a wider range of combinations between them and, on the other hand, other pigments can be used for colorimetric formulations. Another development path for future studies is an improved experimental design, in terms of better coverage of the dental color space with the manufactured samples. A proper distribution of the samples within the area of interest of the color space will allow the use of newer, more accurate and reliable predictive methods, such as Fuzzy Logic.

However, one should not underestimate the importance of the exact knowledge of the reflectance spectrum of the samples to be analyzed, since by knowing the reflectance factor values for any wavelength within the visible spectrum (380 n-780 nm) we are able to calculate the values of the chromaticity coordinates under any illuminant (not only the daylight simulator - CIE D65) and for any CIE Colorimetric Standard Observer. These calculations have the potential to provide important additional information, depending on the objective of the study. In this sense, if the study is intended to cover a widely range of colorimetric coordinates under different Standard Illuminants and with different Standard Observers, it is advisable to design predictive models for reflectance factors at each wavelength, which will allow to make various calculations based on the equations provided by the International Commission on Illumination (CIE).

Soft science applications involve so many variables that it is not practical to seek a model which explicitly relates them all. The Multiple Nonlinear Regression is one of the possible solutions and although it is continuously evolving as a statistical modeling technique, there are other fields which can provide also good results, such as principal components regression, maximum redundancy analysis, methods which handle the colinearity in regression, such as the ridge regression[21], or newer methods, such as the neural networks[22]. The neural networks are probably the strongest competitors for MNLR in terms of flexibility and robustness of the predictive models, but they do not explicitly incorporate a linear extraction of latent factors - that is dimension reduction[23].

## Conclusions

Within the framework of this preliminary study, the nonlinear predictive models developed allow the prediction, with a high degree of accuracy, of the reflectance spectrum of the experimental dental resin composites (average error <3.54% across all wavelengths of the visible spectrum). The differences found between the predicted and measured values of the chromatic coordinates are smaller than the acceptability threshold established for this type of materials and, in some cases, are even below the perceptibility threshold. These results open the way for custom design of dental resin composites, with multiple direct and immediate clinical applications, such as the manufacture of dental shade guides, development of new dental materials, and finally, performing dental restorations that perfectly match the color of their surrounding dental structures. However, before bringing these materials to the clinic, the present study has to be complemented with studies on other physical and chemical properties of the material, such as polymerization shrinkage, hardness, wear resistance, degree of polymerization, temporal and thermo chromatic stability, etc.

## Competing interests

The authors declare that they have no competing interests.

## Authors' contributions

All authors contributed equally to this work.

## Declarations

Publication of this article was supported by Spanish Ministry of Economy and Competitiveness MAT2013-43946-R Grant.

This article has been published as part of *BioMedical Engineering OnLine *Volume 14 Supplement 2, 2015: Select articles from the 2nd International Work-Conference on Bioinformatics and Biomedical Engineering (IWBBIO 2014). The full contents of the supplement are available online at http://www.biomedical-engineering-online.com/supplements/14/S2.
